# Posterior stabilization with polyetheretherketone (PEEK) rods and transforaminal lumbar interbody fusion (TLIF) with titanium rods for single-level lumbar spine degenerative disease in patients above 70 years of age

**DOI:** 10.1007/s00402-022-04448-8

**Published:** 2022-05-05

**Authors:** M. Kamenova, E. Li, J. Soleman, O. Fiebig, A. Mehrkens, S. Schaeren

**Affiliations:** 1 Department of Spine Surgery, University Hospital of Basel, Spitalstrasse 21, 4051 Basel, Switzerland; 2 Department of Neurosurgery, University Hospital of Basel, Basel, Switzerland; 3 Department of Orthopedic Surgery and Traumatology, University Hospital of Basel, Basel, Switzerland; 4 Faculty of Medicine, University of Basel, Basel, Switzerland

**Keywords:** TLIF, PEEK, Lumbar spine, Interbody fusion, Posterolateral fusion, Elderly patients

## Abstract

**Background:**

Given the lack of guidelines regarding the operative management of elderly patients needing lumbar spine fusion for degenerative disease, it is often difficult to balance between invasiveness respecting the fragile spine and geriatric comorbidities.

**Aim:**

To compare reoperation rates and clinical outcome in patients above 70 years of age undergoing Transforaminal Lumbar Interbody Fusion (TLIF) with titanium rods or posterior stabilization with Polyetheretherketone (PEEK) rods for the treatment of one-level lumbar spine degenerative disease.

**Methods:**

Retrospective review of baseline characteristics, reoperation rates as well as the clinical and radiological outcomes of patients, older than 70 years, undergoing posterolateral fusion with PEEK rods (*n* = 76, PEEK group) or TLIF with titanium rods (*n* = 67, TLIF group) for a single-level lumbar degenerative disease from 2014 to 2020. Additional subanalysis on the patients above 80 years of age was performed.

**Results:**

Our results showed similar reoperation rates and outcomes in the TLIF and PEEK groups. However, intraoperative blood loss, administration of tranexamic acid, and operation time were significantly higher in the TLIF group. In patients older than 80 years, reoperation rates at first follow-up were significantly higher in the TLIF group, too.

**Conclusion:**

According to our results, posterior stabilization with PEEK rods is less invasive and was associated with significantly lower blood loss, administration of blood products and shorter operation time. Moreover, in patients above 80 years of age reoperations rates were lower with PEEK rods, as well. Nevertheless, the benefits of PEEK rods for foraminal stenosis still have to be investigated.

## Introduction

Lumbar spine degenerative disease and instability are common causes of intractable pain due to abnormal motion and neural compression [1]. In these cases, pedicle screws and rod instrumentation are widely accepted procedures for achieving stabilization and fusion in the affected segments [2–4].

One of the most popular surgical techniques for the treatment of spondylolisthesis, degenerative disc disease, pseudarthrosis, adjacent segment degeneration (ASD) or degenerative scoliosis is the transforaminal lumbar interbody fusion (TLIF) [5–8]. The advantages of placing an interbody graft include higher arthrodesis rates, off-loading posterior instrumentation and restoring of disc space height and lordosis [5]. Moreover, TLIF has been shown to have advantages in complication rates, operation times and blood loss compared to the traditional posterior lumbar interbody fusion (PLIF) for the treatment of degenerative spondylolisthesis [9]. However, experimental studies have suggested that pedicle-screw-based rigid rod systems, e.g. titanium combined with TLIF decreased the natural intervertebral range of motion (ROM) in the index segment which might lead to an abnormal change of load transfer and thus, accelerates adjacent segment degeneration [10–13]. To overcome these disadvantages, posterior lumbar rods made of polyetheretherketone (PEEK), which seems to be a semirigid alternative to titanium and its alloys, have been introduced and showed promising results [1, 4]. However, data on the use of PEEK rods are scarce, so the evidence is not strong enough to definitely confirm a better outcome [14].

As the geriatric population currently rises, more and more elderly patients present with degenerative lumbar spine disease requiring fusion. According to the literature perioperative complications, higher morbidity and mortality have been reported in this population [15–18] with traditional TLIF and PLIF techniques. Posterolateral semirigid fusion via PEEK rods, without using interbody support might be a valid, less invasive alternative for the aged fragile spine.

To date, evidence-based recommendations focusing on the optimal surgical technique in elderly patients are lacking. With the present study, we aim to compare retrospectively reoperation rates and clinical outcome in patients above the age of 70 years with one-level lumbar spine degenerative disease treated by TLIF with titanium rods or posterior stabilization with PEEK rods.

## Methods

We retrospectively reviewed all consecutive patients, older than 70 years, undergoing posterior stabilization with PEEK rods or TLIF with titanium rods for a single-level lumbar degenerative disease from March 2014 to July 2020 at our institution. Patients who had previous operations at the same or adjacent level were excluded from the study. 76 patients (69.7% females) were included in the “PEEK group” and 67 patients (52.2% females) were included in the “TLIF group” (of these, 3 patients received a minimally invasive (MIS) TLIF). The operative technique was based on the decision of the surgeon in charge since established guidelines especially for the use of PEEK rod systems are lacking.

## Surgical techniques

Patients in the TLIF group were operated in a prone position using a midline incision. Pedicle screws (Expedium®, DePuy Synthes Companies, J&J Medical Devices) were inserted into the pedicles at the appropriate levels. The facet joint on one side was removed partially or completely and the nerve roots were identified and decompressed. In the case of a bilateral pathology, decompression was also performed on the contralateral side. A microdiscectomy was performed and the vertebral endplates were carefully prepared to facilitate fusion. Autologous bone mixed with Grafton™ DBM was packed into the disc space. An interbody cage was filled with bone and inserted into the disc place. The screws were then connected by titanium rods. A mix of autologous bone and Grafton™ DBM was placed over the facet joints and transverse processes (posterolateral fusion) in 36 (53.7%) patients, while in 31 (46.3%) patients only autologous bone was used for the posterolateral fusion.

MIS TLIF patients (*n* = 3) were operated through an incision made at the level of the facet joint to be resected in the prone position. Pedicle screws were inserted in a minimal invasive technique, via mini-skin incision under radiologic guidance. Soft tissue was bluntly dissected with dilators and a table-mounted tubular retractor was inserted to visualize the facet joint. The inferior and superior facet were resected using Kerrison Rongeurs to the superior border of the pedicle. The ligamentum flavum was resected from lateral to medial to expose the disc. The exiting nerve was decompressed and discectomy was performed exposing the bony endplates. Autologous bone graft was packed into the anterior portion of the discectomy space. A cage was also filled with autologous bone graft and impacted into the disc space. Additional decompression on the contralateral side was performed if required. The screws were then connected by titanium rods. Local/ autologous bone and Grafton™ DBM were placed over the facet joints and transverse processes (posterolateral fusion).

Patients in the PEEK rod group were also operated through a midline incision in the prone position. Pedicle screws (Expedium®, DePuy Synthes Companies, J&J Medical Devices) were inserted into the pedicles at the appropriate levels. Decompression with/without discectomy was performed. The screws were then connected by PEEK rods. Autologous bone graft and Grafton™ DBM were placed over the facet joints and transverse processes (posterolateral fusion) in 12 patients (15.8%), while in 54 (71.1%) only autologous bone graft was used. In 10 patients (13.2%) no posterolateral fusion was performed.

## Outcomes and statistical analysis

The primary endpoint of the study was reoperation rates due to a new symptomatic pathology at the index or adjacent level. Secondary outcomes were intraoperative blood loss, administration of intraoperative blood products, operation time, clinical outcome, surgical and medical morbidity, and mortality. Additionally, a subanalysis for primary and secondary outcomes in patients older than 80 years was performed.

Mean first clinical and radiological follow-up time was 80 ± 33.1 days in the TLIF group and 73.7 ± 27.8 days in the PEEK rod group, *p* = 0.29. Last clinical and radiological follow-up time on average was 282.6 ± 150.4 days in the TLIF group and 300 ± 134.1 days in the PEEK rod group (*p* = 0.28). Consultations for new symptoms after regular follow-up were also assessed. Screw loosening was defined as radiolucency of 1 mm or more around the implant. Adjacent segment degeneration (ASD) was defined as radiographic degenerative changes at a spinal level immediately cranial or caudal to the site of the previous fusion [19]. Fusion was assessed through plain static X-ray films including anterior–posterior and lateral levels as CT scans are not routinely performed at follow-up at our institution. Two authors (E.L. and M.K.) graded the X-ray films for evidence of high interbody and posterolateral fusion at last follow-up. This was defined as bone bridges at at least half of the fusion area for TLIF, with at least the density achieved at surgery corresponding to a BSF 3 [20], while high fusion in the PEEK rod group was defined as bilateral trabeculated fusion masses similar to a Lenke Grade A [21] and absence of screw loosening. Overall lumbar lordosis (LL) was defined as the angle between the upper plate of the first lumbar and first sacral vertebral bodies. Segmental LL was measured by joining perpendiculars to lines drawn parallel to the upper endplate of the higher index vertebral body and the lower endplate of the lower index vertebral body. All LL parameters were analyzed by E.L. M.K and O.F. separately and checked for interobserver variability.

The study protocol was approved by the local ethics committee (EKNZ, Basel, Switzerland). The correlation between surgery type and outcome measures was analyzed using a contingency table and calculating the Fisher’s exact or the Chi-square test. For all other parameters, contingency tests were done using Fisher’s exact test, while all other calculations were done using Mann–Whitney-U tests. All statistical analyses were done using SPSS Statistics Version 21.0 (IBM Corp, 2012). A p value of < 0.05 was considered significant.

## Results

### Baseline characteristics

Age, sex, comorbidities, laboratory findings, ASA score, BMI, and presenting symptoms were collected retrospectively. Radiological findings such as diagnosis, spondylolisthesis grade according to the Meyerding classification, preoperative segmental and overall lumbar lordosis, and level of pathology were analyzed, as well. While patients were well matched concerning BMI, ASA score and comorbidities, significant differences were found in some clinical and radiological findings (Table 1).

**Table 1:** Baseline characteristics

	TLIF group (*n* = 67)	PEEK group (*n* = 76)	*p* value
Sex (f) (%)	35 (52.2)	53 (69.7)	**0.02**
Age (y) (mean ± SD)	76.6 ± 4.6	78.3 ± 5.1	**0.04**
BMI (mean ± SD)	26.3 ± 4.6	26.1 ± 4.5	0.56
ASA Score			0.70
1	1 (1.5)	2 (2.6)	
2	36 (53.7)	36 (47.4)	
3	30 (44.8)	38 (50)	
Comorbidities *n* (%)			
Diabetes mellitus	9 (13.4)	6 (7.9)	0.37
Osteoporosis	10 (14.9)	10 (13.2)	0.81
Osteoporosis treatment (yes)	5 (7.5)	5(6.6)	0.56
Hb preop. (g/L) (mean ± SD)	138.61 ± 14.87	132.0 ± 13.46	**0.01**
Hb preop. (d) (mean ± SD)	1.4 ± 1.1	1.6 ± 1.3	0.26
Diagnosis *n* (%)			
Central stenosis and olisthesis	30 (44.8)	66 (86.8)	**0.00**
Joint cyst	1 (1.5)	5 (6.6)	
Foraminal stenosis and olisthesis	21 (31.3)	2 (2.6)	
Foraminal stenosis without olisthesis	9 (13.4)		
Disc herniation	2 (3)		
Central stenosis and foraminal stenosis and olisthesis	3 (4.5)	3 (3.9)	
Meyerding grade *n* (%)			
0	10 (14.9)	2 (2.6)	**0.03**
1	49 (73.1)	66 (86.8)	
2	8 (11.9)	8 (10.5)	
Level *n* (%)			
L1/2	1 (1.5)	3 (3.9)	**0.00**
L2/3	1 (1.5)	1 (1.3)	
L3/4	7 (10.4)	9 (11.8)	
L4/5	40 (59.7)	63 (82.9)	
L5/S1	18 (26.9)		
Preop. segmental lordosis deg.* (mean ± SD)	19 ± 8.16	17.9 ± 7.95	0.62
Preop. overall lordosis deg.** (mean ± SD)	45.0 ± 11.38	48.6 ± 12.69	0.09
Preop. symptoms *n* (%)			**0.00**
Claudication spinalis	28 (41.8)	56 (73.7)	
Radicular pain	34 (50.7)	12 (15.8)	
Motor deficits	1 (1.5)	3 (3.9)	
Cauda equina syndrome	1 (1.5)		
Claudication spinalis and radicular pain	3 (4.5)	5 (6.6)	
Back pain	40 (59.7)	42 (55.3)	0.62

*f* female, y years, *n* number, *hb* hemoglobin, *SD* standard deviation

p value < 0.05: significant

Bold: significant

*Measured by joining perpendiculars to lines drawn parallel to the upper endplate of the higher index vertebral body and the lower endplate of the lower index vertebral body

**The angle between the upper plate of the first lumbar and first sacral vertebral bodies

### Primary outcome measures

#### Reoperation rates at first regular follow-up

Reoperation rates due to a new symptomatic pathology at adjacent or index levels at first follow-up were 9% (*n* = 6) in the TLIF group and 5.3% (*n* = 4) in the PEEK rod group, showing no statistical difference, *p* = 0.56. Reasons for surgery were wound healing disorders (4.5%, *n* = 3 vs. 2.6%, *n* = 2), ASD (0 vs. 2.9%, *n* = 1), insufficiency fractures (3%, *n* = 1 vs. 3%, n = 1) and pedicle screw loosening (4.8%, *n* = 1 vs. 4.3%, *n* = 1) in the TLIF and PEEK rod groups, respectively, *p* = 0.37. Furthermore, one case of symptomatic cage subsidence needing revision surgery occurred in the TLIF group (Table 3).

#### Reoperation rates at last follow-up

At last follow-up rates of revision surgery did not differ significantly with 9% (*n* = 6) vs. 6.6% (*n* = 5), *p* = 0.60 in the TLIF and PEEK rod groups, respectively. Of these, reasons for revision surgery were ASD (6.7%, *n* = 4 vs. 4.3%, *n* = 3), insufficiency fractures (1.7%, *n* = 1 vs. 1.5%, *n* = 1), pedicle screw loosening (0 vs. 1.5%, *n* = 1), delayed union (1.7%, *n* = 1 vs. 0) in the TLIF and PEEK rod groups, respectively (*p* = 0.17) (Table 3).

After complete regular follow-up, 3 patients (4.5%) in the TLIF group and 5 patients (6.6%) in the PEEK rod group presented with new symptoms, (*p* = 0.72). Of these, only one patient in the PEEK rod group was treated surgically for symptomatic ASD (*p* = 0.62). The average times of new presentation, symptoms, and imaging findings are described in Table 3.

##### Secondary outcome measures

#### Intraoperative findings, surgical and medical complications

TLIF patients received significantly more often tranexamic acid (Cyclokapron) intraoperatively, compared to the PEEK rod group (50.7% (*n* = 34) vs. 30.3% (*n* = 23) respectively, *p* = 0.04). Blood loss was significantly higher in the TLIF group (479.10 ± 273.88 ml vs. 337.11 ± 180.50 ml, *p* < 0.001).

Furthermore, operation time was significantly longer in the TLIF group (197.43 ± 61.23 vs. 145.96 ± 54.23 min), respectively, *p* < 0.001. Surgical and medical complications, as well as hospitalization time were similarly distributed in both groups and are presented in Table 2. In our cohorts, no mortality occurred.

**Table 2:** Intraoperative findings, surgical and internistic complications

	TLIF group (*n* = 67)	PEEK group (*n* = 76)	*p* value
Intraop. blood products *n* (%)	35 (52.2)	24 (31.6)	**0.02**
Intraop. blood products type *n* (%)			
Tranexamic acid (Cyclokapron)	34 (50.7)	23 (30.3)	**0.04**
RBC and Cyclokapron	1 (1.5)		
Blood loss (ml) (mean ± SD)	479.10 ± 273.88	337.11 ± 180.50	**0.00**
Hb postop. (g/L) (mean ± SD)	108 ± 15.18	107.27 ± 13.74	0.42
Hb postop. (d) (mean ± SD)	2 ± 1.3	2 ± 1.1	0.54
OR time (min) (mean ± SD)	197.43 ± 61.23	145.96 ± 54.23	**0.00**
Surgical complications during hospitalization yes (%)	3 (4.5)	4 (5.3)	1
Surgical complications needing revision surgery *n* (%)	1 (1.5)	1 (1.3)	1
Surgical complication type *n* (%)	3 (2.1)	4 (2.8)	0.57
EDH	1 (33.3)	2 (50)	
Dural tear	1 (33.3)	2 (5)	
Wound healing disorder	1 (33.3)		
Internistic complications yes (*n*)	10 (14.9)	10 (13.2)	0.81
Internistic complications (d. postop.)	5.2 ± 3.5	4.9 ± 3.1	
Type of internistic complication *n* (%)			0.43
Postop delirium	1 (10)		
Urinary infection	2 (20)	4 (40)	
DVT	1 (10)		
Pneumonia		1 (50)	
Bladder dysfunction	5 (50)	2 (20)	
Cardiac decompensation		1 (50)	
Stroke		1 (50)	
ileus	1(10)	1 (50)	
Hospitalization time (*d*)	10.7 ± 5.15	11.3 ± 4.45	0.12

*n* number, *hb* hemoglobin, *RBC* red blood cells, *min* minutes, *d* day, *EDH* epidural hematoma, *DVT* deep vein thrombosis, *SD* standard deviation

*p* value < 0.05: significant

Bold: significant

#### Clinical outcome

Clinical outcomes at discharge did not differ significantly between the groups with the majority of the patients feeling “better” (97% (*n* = 65) in the TLIF group and 96.1%, (*n* = 73) in the PEEK group, *p* = 0.75). At first and last follow-up clinical outcome was comparable in both groups with patients feeling “better” in 83.3% (*n* = 55) and 81.3% (*n* = 61) within the TLIF group and 88.3% (*n* = 53) and 86.6% (*n* = 58) within the PEEK rod group (Table 3).

**Table 3:** Clinical and radiological outcome

	TLIF group (*n* = 67)	PEEK group (*n* = 76)	*p* value
Symptoms at discharge *n* (%)			
Better	65 (97)	73 (96.1)	0.75
Same	2 (3)	2 (2.6)	
Discharge destination *n* (%)			
Home	48 (71.6)	50 (65.8)	0.48
Rehabilitation	19 (28.4)	26 (34.2)	
Type of imaging at 1st FU *n* (%)			
X-Ray	55 (88.7)	58 (82.9)	0.37
MRI	1 (1.5)		
X-ray and MRI	3 (4.8)	7 (10)	
X-ray and CT scan	2 (3.2)	3 (4.3)	
X-ray and MRI and CT scan	1 (1.5)		
Imaging finding at 1st FU *n* (%)			
Normal	54 (80.6)	62 (89.9)	0.17
Pedicle screw loosening	1 (1.5)	2 (2.9)	
ASD	0	2 (2.9)^a^	
Cage subsidence	4 (6.5)		
Insufficiency fracture	3 (4.8)	3 (4.3)	
Symptoms at first FU *n* (%)			
Better	55 (83.3)	61 (81.3)	0.95
Worse	4 (6.1)	5 (6.6)	
Same	7 (10.6)	9 (12)	
Revision surgery at first FU yes (%)	6 (9.0)	5 (6.6)	0.56
Reason for revision surgery at first FU *n* (%)			
Wound healing disorder	3 (4.5)	2 (2.6)	0.37
ASD	0	1 (1.4)	
Insufficiency fracture	1 (1.5)	1 (1.4)	
Pedicle screw loosening	1 (1.5)	1 (1.4)	
Cage subsidence	1 (1.5)		
Type of imaging at last FU n (%)			
X-ray	50 (84.7)	56 (83.6)	0.74
X-ray and MRI	6 (10.2)	7 (10.4)	
X-ray and MRI and CT scan	3 (4.8)	4 (5.3)	
High fusion *n* (%)	46 (78%)	46 (68.7)	**0.04**
Imaging finding at last FU *n* (%)			0.35
Normal	48 (81.4)	57 (85.1)	
Pedicle screw loosening	0	1 (1.5)	
ASD	6 (10.2)	8 (11.9)	
Cage subsidence	3 (5.1)		
Insufficiency fracture	1 (1.7)	1 (1.5)	
Delayed union	1 (1.7)		
ASD location *n* (%)			
Cranial segment	4 (6.4)	5 (6.6)	0.66
Caudal segment	2 (3.2)	3 (4.3)	
ASD finding *n* (%)			
Disc herniation	1 (1.7)	7 (10.4)	**0.03**
Stenosis	3 (5.1)	1 (1.5)	
Instability	2 (3.2)		
Symptoms at last FU *n* (%)			
Better	53 (88.3)	58 (86.6)	0.65
Worse	3 (5.1)	2 (2.6)	
Same	4 (6.7)	7 (10.4)	
Revision surgery at last FU yes *n* (%)	6 (9.0)	5 (6.6)	0.60
Reason for revision surgery at last FU *n* (%)			
ASD	4 (6.7)	3 (4.3)	0.17
Insufficiency fracture	1 (1.7)	1 (1.5)	
Pedicle screw loosening		1 (1.5)	
Delayed union	1 (1.7)		
Type of revision surgery for ASD *n* (%)			
Decompression	2 (3.2)		0.29
Decompression and stabilization	2 (3.2)	3 (4.3)	
New symptoms after complete FU yes *n* (%)	3 (5.1)	5 (6.6)	0.72
New symptoms d postop. (mean ± SD)	892.3 ± 482.52	1123 ± 601.90	0.66
Symptoms *n* (%)			
Claudicatio	1 (1.7)	2 (2.6)	0.16
Radicular pain		3 (4.3)	
Pseudoradicular pain	2 (3.2)		
New radiological finding			
Normal	1 (1.7)		0.38
ASD	2 (3.2)	5 (6.6)	
ASD location *n* (%)			
Cranial	2 (3.2)	4 (5.3)	1
Caudal		1 (1.5)	
ASD finding *n* (%)			
Disc herniation		2 (2.6)	0.19
Stenosis	1 (1.7)	3 (4.3)	
Instability	1 (1.7)		
Revision surgery yes (%)		1 (1.3)	0.62
Revision surgery type			
Decompression		1 (1.3)	
Postop. segmental lordosis deg.* (mean ± SD)	20.29 ± 7.68	18.67 ± 8.0	0.39
Postop. overall lordosis deg.** (mean ± SD)	45.6 ± 10.97	49.5 ± 12.6	0.07

*f* female, *y* years, *n* number, *hb* hemoglobin, *ASD* adjacent segment degeneration, *FU* follow-up

SD: standard deviation

*p* value < 0.05: significant

Bold: significant

*Measured by joining perpendiculars to lines drawn parallel to the upper endplate of the higher index vertebral body and the lower endplate of the lower index vertebral body

**The angle between the upper plate of the first lumbar and first sacral vertebral bodies

### Subanalysis on the patients older than 80 years

Seventeen patients (25.4%) in the TLIF group and 32 patients (42.1%) in the PEEK rod groups were older than 80 years. Reoperations at first follow-up were performed in 17.6% (*n* = 3) of the patients in the TLIF group (1 case of wound healing disorder, 1 case of ASD and 1 case of pedicle screw loosening), while in the PEEK rod group none of the patients were reoperated, showing statistical significance (*p* = 0.04).

When comparing secondary outcome measures, blood loss was significantly higher in the TLIF group compared to the PEEK rod group (444.12 ml ± 10.89 vs. 343.75 ml ± 217.29, *p* = 0.04). Moreover, operation time was significantly longer in the TLIF group (188.29 min ± 54.9 vs. 146.43 min ± 59.45, *p* < 0.001).

Baseline characteristics and complete results on the primary and secondary outcomes of the subgroup of patients older than 80 years are presented in Tables 4, 5 and 6.

**Table 4:** Baseline characteristics of patients older than 80 years

	TLIF group (*n* = 17)	PEEK group (*n* = 32)	*p* value
Sex (f) (%)	11 (64.7)	23 (71.9)	0.42
Age (y) (mean ± SD)	82.9 ± 2.7	83.5 ± 2.2	0.30
BMI (mean ± SD)	26.5 ± 5.6	25.4 ± 3.6	0.68
ASA score			
1			0.50
2	5 (29.4)	11 (34.4)	
3	12 (70.6)	21 (65.6)	
Comorbidities *n* (%)			
Diabetes mellitus	2 (11.8)	2 (6.3)	0.62
Osteoporosis	4 (23.5)	6 (18.6)	0.72
Osteoporosis treatment (yes)	1 (5.9)	3 (9.4)	0.36
Diagnosis *n* (%)			**0.00**
Central stenosis and olisthesis	6 (35.3)	29 (90.6)	
Joint cyst		1 (3.1)	
Foraminal stenosis and olisthesis	8 (47.1)		
Foraminal stenosis without olisthesis	2 (11.8)		
Central stenosis and foraminal stenosis and olisthesis	1 (5.9)	2 (6.3)	
Meyerding grade *n* (%)			
0	2 (11.8)		0.14
1	13 (76.5)	28 (87.5)	
2	2 (11.8)	4 (12.5)	
Level *n* (%)			
L1/2	1 (5.9)	1 (3.1)	0.05
L2/3		1 (3.1)	
L3/4	1 (5.9)	5 (15.6)	
L4/5	11 (64.7)	25 (78.1)	
L5/S1	4 (23.5)		
Preop. segmental lordosis deg.* (mean ± SD)	20.15 ± 11.6	16.79 ± 8.30	0.40
Preop. overall lordosis deg.** (mean ± SD)	39.48 ± 12.17	46.24 ± 13.46	0.09
Preop. symptoms *n* (%)			
Claudication spinalis	8 (47.1)	27 (84.4)	**0.00**
Radicular pain	8 (47.1)	1 (3.1)	
Motor deficits		1 (3.1)	
Claudication spinalis and radicular pain Back pain	1 (5.9) 12 (70.6)	3 (9.4) 13 (40.6)	**0.04**

*SD* standard deviation

*p* value < 0.05: significant

Bold: significant

*f* female, *y* years, *n* number, *ASD* adjacent segment degeneration, *FU* follow-up

*Measured by joining perpendiculars to lines drawn parallel to the upper endplate of the higher index vertebral body and the lower endplate of the lower index vertebral body

**The angle between the upper plate of the first lumbar and first sacral vertebral bodies

**Table 5:** Intraoperative findings, surgical and internistic complications in patients older than 80 years

	TLIF group (*n* = 17)	PEEK group (*n* = 32)	*p* value
Intraop. blood products *n* (%)	8 (47.1)	12 (37.5)	0.56
Intraop. blood products type *n* (%)	8 (47.1)	12 (37.5)	0.56
Tranexamic acid (Cyclokapron)			
Blood loss (ml) (mean ± SD)	444.12 ± 10.89	343.75 ± 217.29	**0.04**
Hb postop. (g/L) (mean ± SD)	101.50 ± 17.01	104.63 ± 13.34	0.69
OR time (min) (mean ± SD)	188.29 ± 54.9	146.43 ± 59.45	**0.00**
Surgical complications yes (%)		1 (3.1)	0.66
Surgical complications needing revision surgery *n* (%)		1 (3.1)	0.66
Surgical complication type *n* (%)			
EDH			
Dural tear		1 (3.1)	
Wound healing disorder			
Internistic complications yes (*n*)	4 (23.5)	4 (12.5)	0.42
Internistic complications (d postop.)	4 ± 2.2	4 ± 1.6	0.49
Type of internistic complication *n* (%)			
Postop delirium	1 (5.9)		0.51
Urinary infection	1 (5.9)	2 (6.3)	
DVT	2 (11.8)	2 (6.3)	
Hospitalization time (d) (mean ± SD)	11.6 ± 6.4	11.1 ± 4.9	0.71

*n* number, *hb* hemoglobin, *min* minutes, *d* day, *EDH* epidural hematoma, *DVT* deep vein thrombosis, *SD* standard deviation

*p* value < 0.05: significant

Bold: significant

**Table 6:** Clinical and radiological outcome in patients older than 80 years

	TLIF group (*n* = 17)	PEEK group (*n* = 32)	*p* value
Symptoms at discharge *n* (%)			
Better	16 (94.1)	31 (96.9)	0.58
Same	1 (5.9)	1 (3.1)	
Discharge destination *n* (%)			
Home	8 (47.1)	20 (62.5)	0.38
Rehabilitation	9 (52.9)	12 (37.5)	
Type of imaging at 1st FU *n* (%)			
X-Ray	12 (80)	24 (87.5)	0.10
MRI	1 (5.9)		
X-ray and MRI		4 (14.3)	
X-ray and CT scan	1 (5.9)		
X-ray and MRI and CT scan	1 (5.9)		
Imaging finding at 1st FU *n* (%)			
Normal	11 (73.3)	26 (92.9)	0.20
Pedicle screw loosening	1 (5.9)	1 (3.1)	
ASD	1 (5.9)		
Cage subsidence	2 (13.3)		
Insufficiency fracture	1 (5.9)	1 (3.1)	
Symptoms at first FU *n* (%)			
Better	14 (82.4)	26 (81.3)	0.06
Worse		1 (3.1)	
Same	3 (17.6)	5 (15.6)	
Revision surgery at first FU yes (%)	3 (17.6)		**0.04**
Reason for revision surgery at first FU *n* (%)			
Wound healing disorder	1 (5.9)		0.27
Asd	1 (5.9)		
Pedicle screw loosening	1 (5.9)		
Type of imaging at last FU *n* (%)			
X-ray	11 (84.6)	25 (86.2)	0.82
X-ray and MRI	1 (5.9)	3 (10.3)	
X-ray, MRI and CT scan	1 (5.9)	1 (3.1)	
High fusion	9 (69.2)	19 (65.5)	0.14
Imaging finding at last FU *n* (%)			
Normal	10 (76.9)	26 (89.7)	0.08
Pedicle screw loosening		1 (3.1)	
ASD		2 (6.9)	
Cage subsidence	2 (15.4)		
Insufficiency fracture	1 (5.9)		
ASD location *n* (%)			
Cranial segment		1 (3.1)	
Caudal segment		1 (3.1)	
ASD finding *n* (%)			
Disc herniation		1 (3.1)	
Stenosis		1 (3.1)	
Symptoms at last FU *n* (%)			
Better	12 (92.3)	27 (93.1)	0.67
Worse	1 (5.9)	1 (3.1)	
Same		1 (3.1)	
Revision surgery at last FU yes *n* (%)	1 (5.9)	2 (6.9)	0.73
Reason for revision surgery at last FU *n* (%)			
ASD		1 (3.1)	0.17
Insufficiency fracture	1 (5.9)		
Pedicle screw loosening		1 (3.1)	
Type of revision surgery *n* (%)			
Decompression			
Decompression and stabilization	1 (5.9)	2 (6.9)	
New symptoms after complete FU yes *n* (%)	1 (5.9)	1 (3.1)	0.58
New symptoms after complete FU d postop (mean ± SD)	900	679	0.32
Symptoms *n* (%)			
Radicular pain		1 (3.1)	0.50
Pseudoradicular pain	1 (5.9)		
Radiological finding			
Normal	1 (5.9)		0.50
ASD		1 (3.1)	
ASD location n(%)			
Cranial		1 (3.1)	
Caudal			
ASD finding *n*(%)			
Stenosis		1 (3.1)	
Revision surgery yes (%)		1 (3.1)	0.50
Postop. segmental lordosis deg.* (mean ± SD)	21.39 ± 10.34	18.19 ± 8.35	0.35
Postop. overall lordosis deg.** (mean ± SD)	40.80 ± 12.62	47.47 ± 1310	0.10

*n* number, *ASD* adjacent segment degeneration, *FU* follow-up, *SD* standard deviation

*p* value < 0.05: significant

Bold: significant

*Measured by joining perpendiculars to lines drawn parallel to the upper endplate of the higher index vertebral body and the lower endplate of the lower index vertebral body

**The angle between the upper plate of the first lumbar and first sacral vertebral bodies

## Discussion

Our results showed similar reoperation rates both in the TLIF and PEEK rod groups at the first and last follow-up. Surgical and medical complication rates, as well as clinical and radiological outcome measures did not differ significantly, either. However, intraoperative blood loss, intraoperative administration of tranexamic acid and operation time were significantly higher in the TLIF- compared to the PEEK rod group. When comparing outcomes in the subgroup older than 80 years, blood loss, operation time, but also reoperation rates at first follow-up were significantly higher in the TLIF group.

With modern medicine, the population aged 60 years or older is expected to rise up to 22% by 2050 [22]. This shift will affect spine surgical practice, as well, therefore factors like geriatric comorbidities, minimal invasiveness, and pitfalls of the aging spine will become more and more relevant.

TLIF with titanium rods is a widely accepted fusion technique for patients with degenerative disc disease or adult spinal deformity and has been considered by many as the gold standard for the treatment of these pathologies [23–27]. However, controversial reports concerning outcomes and lack of high-class evidence persist when it comes to fusion procedures in elderly patients. Some authors revealed an increased rate of major medical complications and mortality following 1 to 2 level lumbar posterolateral fusion [28]. Others found that lumbar spinal fusion in patients above the age of 60 years is associated with a higher incidence of postoperative morbidity and mortality than spine surgery without fusion [29]. On the other hand, it has been reported that age was not associated with complications nor predictive of functional outcome in patients undergoing multilevel TLIF [15, 30]. As rigid systems may contribute to stress shielding in the anterior column and may lead to more ASD, PEEK rods have been introduced as providing a modulus of elasticity close to cancellous bone that may have a better risk profile [31]. A prospective study on the Dynesys system showed no progression of spondylolisthesis after 2 years and stable implants [32]. Other small cohort studies on PEEK rod systems suggest that they provide a safe and effective use with comparable stability to titanium rods but decreased risk of failure [31].

Our retrospective analysis demonstrated similar results in terms of reoperation rates and clinical outcome with both techniques in patients older than 70 years. Biomechanical studies suggested that PEEK rods have comparable intervertebral stability with titanium rods, but lower risk of screw mobilization in poor bone, an increased anterior physiological load and a reduced stress on the bone-screw interface which might reduce ASD [31]. However, according to our results, radiological outcomes like ASD, implant failure or insufficiency fractures did not differ among the groups. Radiological ASD was noted in 10.2% (*n* = 6) and 11.9% (*n* = 9) in the TLIF and PEEK rod groups respectively, consistent with the range described in the literature [33]. Interestingly, adjacent disc herniations were significantly more common in the PEEK rod group, while adjacent spinal stenosis was more common in the TLIF group. At the same time, evidence for high fusion was significantly more common in the TLIF group, although in the PEEK rod group fusion was performed in 86.9% (*n* = 66) of the cases. The reasons for the different types of ASD remain unclear. Possible, better fusion in the index segment in the TLIF group might cause more stress in all adjacent segment elements (facet joints, ligaments and disc) which may accelerate spinal stenosis. With PEEK rods and less solid fusion, mostly adjacent discs seem to be affected resulting in the above-mentioned type of ASD.

According to our results, the advantage of a posterior stabilization via PEEK rods compared to TLIF seems to be the lower invasiveness, shorter OR time, as well as lower blood loss and administration of blood products. These findings might be crucial especially for geriatric patients, although in our cohorts no unfavorable consequences in terms of clinical outcome were detected. Moreover, posterior stabilization with PEEK rods is a more cost-effective procedure as expensive cages are not needed and OR time is shorter. As it comes to the older patients (> 80 years), in our cohort the benefits of the PEEK rod systems seem to be more evident as not only blood loss and operation time were significantly higher in the TLIF group, but also reoperation rates in first follow-up (3 (17.6%) vs. 0 cases). This might indicate that choosing a PEEK rod stabilization might be also reasonable when it comes to > 80 years old patients. However, definitive conclusions cannot be drawn because of the rather small number of patients in both groups. Indication for PEEK rods in our cohort was mostly central stenosis and olisthesis (86.6%). The general question still remains if simple decompression would not be sufficient especially for grade I olisthesis. A recently published meta-analysis showed no advantage of one procedure over the other. The decompression-only cohort had fewer complications but a higher revision rate [34].

## Limitations

This retrospective study is subject to all the limitations of data collection inherent in such works (e.g. missing information on parameters of sagittal balance such as pelvic incidence/ pelvic tilt or standard questionnaires for clinical outcome).

We recognize that our results might be biased by the fact that the treating surgeon chose the surgical technique. Significantly more patients with central stenosis and olisthesis received a PEEK rod stabilization, while significantly more patients with foraminal stenosis were treated with a TLIF. Nevertheless, these were all patients over 70 years with one-level lumbar spine degeneration and first-time surgery. The constellation of both cohorts depicts the reality of spine surgery as guidelines are lacking and to our knowledge this is the first study to compare both techniques in the geriatric population. A prospective randomized trial is mandatory to overcome the current lack of guidelines and is planned at our institution.

## Conclusion

According to our results, both TLIF with titanium rods and posterior stabilization with PEEK rods seem to be valid and safe surgical techniques for single-level lumbar spine degenerative disease in patients above 70 years of age. However, posterior stabilization with PEEK rods is less invasive and was associated with significantly lower blood loss, administration of blood products and shorter operation time. Moreover, in patients above 80 years of age reoperations rates were lower with PEEK rods, as well. Nevertheless, the benefits of PEEK rods for foraminal stenosis still have to be investigated.
